# Methodological lineages of concept analysis and mapping of reporting elements in nursing and health-related disciplines: A scoping review protocol

**DOI:** 10.1371/journal.pone.0361727

**Published:** 2026-10-30

**Authors:** Sijing Peng, Min Wang, Peili Xu, Xiang Wang, Ting Zheng, Min You, Jiejie Ge

**Affiliations:** 1 School of Nursing, Anhui University of Chinese Medicine, Hefei, Anhui, China; 2 Department of Nursing, Huangshan City People’s Hospital, Huangshan, Anhui, China; 3 Nursing Department, Hefei First People’s Hospital, Hefei, Anhui, China; 4 The Second Affiliated Hospital of Anhui University of Chinese Medicine, Hefei, Anhui, China; 5 Key Laboratory of Geriatric Nursing and Health, Anhui University of Chinese Medicine, Hefei, Anhui, China; King Khalid University, SAUDI ARABIA

## Abstract

**Introduction:**

Concept analysis is widely used in nursing and health-related disciplines to clarify ambiguous constructs, refine disciplinary language and support theory development, instrument construction, education and practice. However, concept analysis is methodologically plural rather than a single uniform method. Different approaches vary in their assumptions, data sources, analytic steps and expected outputs.

**Aims:**

This scoping review will identify methodological lineages of concept analysis and map reporting elements across nursing and health-related disciplines. It will examine both the cross-lineage applicability and the empirical distribution of candidate reporting elements.

**Methods:**

The review will follow Joanna Briggs Institute guidance for scoping reviews and will be reported according to the PRISMA extension for Scoping Reviews. Ten English- and Chinese-language databases will be searched from inception to the final search date, supplemented by targeted citation searching for foundational methodological sources. Two reviewers will independently screen records after calibration on a purposively enriched set of 25 records containing likely inclusions, likely exclusions and borderline records. Formal screening will begin after both overall raw agreement and positive agreement for the include/uncertain decision reach at least 75%; raw agreement and Cohen’s kappa will be reported before consensus. Data will be charted using a piloted form and synthesised descriptively through deductive-inductive framework analysis. Reporting elements will be mapped by both methodological applicability and empirical distribution.

**Conclusion:**

The review will provide a structured account of concept analysis methods and reporting elements. It represents an evidence-mapping and candidate-item generation stage and will not itself produce a formal reporting guideline. The protocol is registered on the Open Science Framework: https://osf.io/xru7f.

## Introduction

Concept analysis is widely recognised in nursing scholarship as a methodological approach for clarifying ambiguous constructs, strengthening disciplinary language and supporting theory development, research and practice [1–4]. Rather than functioning only as an abstract theoretical exercise, concept analysis helps refine conceptual boundaries, improve interpretive precision and support more coherent knowledge development in nursing [1–4].

Concept analysis in nursing is methodologically plural. Contemporary scholarship continues to draw on Walker and Avant’s approach, Rodgers’ evolutionary approach, hybrid models and other derivative or pragmatic methods, each of which differs in philosophical assumptions, data sources, analytic procedures and expected outputs [1,3–5]. This plurality has allowed concept analysis to be adapted to diverse clinical, organisational, educational and theoretical questions, but it also complicates transparent and comparable reporting.

Recent publications indicate that concept analysis remains active across contemporary nursing topics and settings. Examples include decision fatigue in nursing, organisational resilience in nursing, nursing followership, expertise among practising clinical nurses and adult social care nursing [6–10]. These studies illustrate the continued use of concept analysis across clinical, organisational, leadership and social care contexts, and show that concept analysis remains relevant to current nursing knowledge development.

However, concept analysis has also been subject to persistent methodological criticism. Longstanding critiques have noted that some studies oversimplify procedural steps, inadequately justify methodological choices or underreport the processes through which attributes, antecedents, consequences and related elements are derived [11]. More recently, a reporting-focused systematic review in nursing synthesised 111 concept analysis papers and identified 66 candidate reporting items, showing that methodological transparency, data-source reporting, analytic procedures and ethics-related content remained unevenly reported [12]. More broadly, transparent and accurate reporting is central to the reliability, utility and impact of health research, and reporting guidance should be grounded in the structure of the methods it is intended to support [13,14].

Despite these developments, no dedicated scoping review has yet mapped concept analysis methodological lineages together with their reporting elements across nursing and related health disciplines. Existing reporting-focused synthesis has been valuable, but it was not designed to compare methodological traditions systematically or to distinguish cross-lineage applicability from method-specific reporting expectations [12]. The 66 candidate reporting items identified by Nashwan et al [12] will therefore be used in the present review as a traceable initial deductive candidate pool rather than adopted as a reporting standard; lineage-based mapping and inductive coding will be used to examine, harmonise and extend this pool. A scoping review is therefore warranted to identify the range of concept analysis approaches in use, map how reporting elements are distributed within and across methodological lineages, and generate an evidence base for possible future reporting-guideline development in nursing.

Accordingly, this protocol describes a scoping review that will identify methodological lineages of concept analysis and map their reporting elements across nursing and health-related disciplines. Specifically, the review will identify concept analysis methods, compare their theoretical assumptions and analytic processes, map the occurrence and applicability of reporting elements, and generate an initial evidence-informed set of candidate cross-lineage and method-specific items. Formal guideline development, stakeholder consensus, Delphi procedures and pilot testing are outside the scope of this review and would require a separate protocol.

## Materials and methods

### Study design and registration

A scoping review was chosen because the purpose of this study is to map the breadth of concept analysis methodologies used in nursing and health-related disciplines and to examine how these approaches are reported. The review addresses broad exploratory questions about methodological lineages, reporting elements and variation in practice, rather than a narrowly focused question about intervention effects or effect estimates. A scoping review is therefore more appropriate than a systematic review for this purpose [15–18].

The protocol has been developed primarily in accordance with current Joanna Briggs Institute guidance for scoping review protocols and updated methodological guidance for the conduct of scoping reviews [16,17]. PRISMA-P was used only as a supplementary protocol-reporting checklist where applicable [19]. The completed review will be reported in accordance with PRISMA-ScR [15], and final search reporting will follow PRISMA-S [20]. The protocol has been registered on the Open Science Framework (https://osf.io/xru7f).

### Review questions

Guided by the Population-Concept-Context (PCC) framework recommended for scoping reviews [16,17], the review will address the following questions:

- What concept analysis methods have been used in nursing and health-related disciplines?
- How do these methods differ in their theoretical assumptions, data sources, analytic processes and expected outputs?
- How do candidate reporting elements vary in occurrence within methodological lineages, and how broadly are they observed across eligible methodological lineages?
- Which reporting elements are conceptually applicable across eligible methodological lineages, which are lineage-specific, and which remain unclear or contested?

The PCC framework and operational review scope are summarised in Table 1.

**Table 1:** Population-Concept-Context (PCC) framework and operational review scope.

PCC component	Operational definition	Eligibility boundary
Population / sources	Published sources rather than human participants.	Methodological papers, applied concept analyses, comparative or critical discussions, and foundational methodological monographs or book chapters.
Concept	Concept analysis lineages, methodological characteristics, analytic processes and reporting elements.	The source must explicitly describe, apply, compare or critically discuss a concept analysis method or its reporting.
Context	Nursing and health-related disciplines relevant to nursing knowledge development.	English- or Chinese-language sources from database inception; relevance to nursing must be explicit in the source, venue, authorship or stated application.

### Eligibility criteria

#### Population or sources.

This review does not focus on human participants. The unit of inclusion will be published sources that explicitly describe, apply, compare or critically discuss concept analysis methods, including foundational methodological monographs or book chapters when they constitute an original or authoritative account of a methodological lineage.

#### Concept.

The core concepts of interest are concept analysis methods or methodological lineages, methodological characteristics of concept analysis and reporting elements used to describe or present concept analysis studies. Sources will be eligible if they provide explicit information about the methodological approach to concept analysis, its underlying framework, analytic steps, outputs or reporting features.

#### Context.

The review will focus on nursing and health-related disciplines. Health-related disciplines will include midwifery, rehabilitation, public health, allied health and health services research when the source has an explicit methodological connection to nursing knowledge development, education, research or practice. Relevance will be established through at least one prespecified non-circular indicator: publication in a nursing or allied-health venue; nursing or allied-health authorship or setting; or explicit discussion of nursing application or relevance. Citation chaining may identify candidate sources, but citation alone will not establish nursing relevance. Ambiguous cases will be independently assessed by two reviewers and adjudicated by a third reviewer.

#### Types of sources.

The review will consider methodological papers describing concept analysis approaches; empirical or theoretical studies that explicitly apply a named concept analysis method; comparative or critical discussions of concept analysis approaches; and foundational methodological monographs or book chapters that provide an original or substantive account of a lineage. Peer-reviewed journal sources will constitute the primary evidence base. Targeted supplementary searching will be used to locate foundational books and chapters. Conference abstracts, editorials, news items, book reviews, non-scholarly websites and sources without sufficient methodological detail will be excluded. Doctoral dissertations will also be excluded to maintain a consistent peer-reviewed published evidence base and a feasible publication-type boundary; this exclusion is not based on an assumption that dissertations lack methodological detail.

#### Language.

English-language and Chinese-language sources will be included.

### Information sources and search strategy

The following electronic databases will be searched from inception to the date of the final search: PubMed/MEDLINE, Embase, CINAHL, Scopus, Web of Science, PsycINFO, CNKI, Wanfang Data, VIP Information and China Biology Medicine. These sources were selected to ensure broad coverage of nursing, health sciences, allied health, methodological literature and Chinese-language scholarship.

The search strategy will be developed iteratively using database-specific controlled vocabulary and fielded free-text terms for concept analysis, named methodological lineages and disciplinary context. Before final execution, two review-team members who did not draft the initial strategy will independently examine the PubMed strategy and its database translations against the PRESS 2015 domains as an internal quality-control checklist [21]. Because an independent information specialist is not available, this internal check will not be described as formal PRESS peer review. Known seed sources will be used to test recall, and all syntax changes and search versions will be documented. Title-only and major-topic restrictions will not be applied routinely because they could reduce sensitivity; proximity operators will be used only where supported by the database platform and shown to improve retrieval. Search strategies and pilot counts are provided in S1 Appendix. Across the ten database-specific pilot searches conducted on 5 May 2026, the final combination steps returned 22,621 records in aggregate before de-duplication. This is a provisional cross-database retrieval count and must not be interpreted as the number of unique, eligible or included sources. The final searches will be re-run or updated before formal screening.

All records identified by the final searches will undergo de-duplication and the prespecified two-stage screening process. All eligible methodological, foundational, comparative and critical sources will be retained. The feasibility contingency will be considered only after full-text eligibility assessment and preliminary lineage assignment; it therefore will not reduce the title/abstract or full-text screening workload and is intended solely to reduce formal charting and verification burden. After the 8–10-source charting pilot, the mean combined time required for charting and verification per source will be used to project the total workload for all eligible applied concept analyses. Sampling will be activated if the projected charting-and-verification workload exceeds 160 combined reviewer-hours, which represents the prespecified maximum combined reviewer capacity allocated to formal charting and verification within the planned review timeline. If activated, 50% of eligible applied concept analyses within each eligible methodological lineage will be selected by reproducible lineage-stratified random sampling, rounded up to the next whole source. A minimum of three eligible applied concept analyses will be retained per lineage where at least three are available; when fewer than three eligible applied concept analyses are available within a lineage, all will be retained. All sources in rare or emerging lineages will also be retained in full. The sampling frame, lineage allocation, sampling fraction, random seed and rationale will be frozen and recorded in the OSF amendment log before sampled charting begins, and no reporting-element data will be used to determine sampling.

Grey literature will not be searched systematically because the review is designed around a reproducible primary evidence base of peer-reviewed published sources, supplemented selectively by authoritative lineage-defining methodological sources. Systematic grey-literature retrieval across two languages and multiple health disciplines would substantially expand the review scope and introduce heterogeneous source types beyond this prespecified boundary. To reduce the risk of missing lineage-defining sources, reference lists and forward citations of included sources and key methodological papers will be examined, and targeted searches by method name and key methodological author will be undertaken to identify foundational monographs or book chapters. Exclusion of other grey literature may underrepresent unpublished or locally disseminated reporting practices and will be considered when interpreting the findings.

### Study selection

All identified citations will be imported into reference management software and de-duplicated before screening. Screening will proceed in two stages. Before title and abstract screening, two reviewers will independently assess a purposively enriched set of 25 records containing likely inclusions, likely exclusions and borderline or uncertain records. Formal screening will begin after both overall raw agreement and positive agreement for the include/uncertain decision reach at least 75%; otherwise, the eligibility criteria will be clarified and calibration will be repeated with a new enriched set. Cohen’s kappa will also be calculated and interpreted descriptively rather than used as the sole screening gate. Before full-text screening, the reviewers will independently assess 5–10 purposively selected boundary cases. All records at both stages will then be assessed independently by two reviewers. Raw agreement and Cohen’s kappa will be calculated before consensus for the calibration exercises and for each completed screening stage. Disagreements will be resolved through discussion and, where necessary, adjudicated by a third reviewer. Reasons for full-text exclusion will be documented. Agreement statistics will be interpreted descriptively and will not replace reasoned eligibility decisions.

The study selection process will be summarised in a PRISMA-ScR flow diagram (**S1 Fig**).

### Data charting

Data will be charted using a structured extraction form developed for this review. Before formal charting, two reviewers will independently pilot the form on 8–10 purposively selected sources representing different methodological lineages and source types. They will compare extracted categorical and free-text data, refine variable definitions, record the charting-and-verification time needed for the feasibility projection, and freeze the first operational codebook before full charting. After full-text eligibility assessment and preliminary lineage assignment, the projected workload will be compared with the prespecified activation threshold described above. If the feasibility contingency is activated, sampling status, lineage stratum, sampling fraction and randomisation records will be retained in the charting file. After calibration and any prespecified sampling decision, one reviewer will chart the retained sources and a second reviewer will verify all extracted data against the source. Agreement on categorical items in the calibration subset will be summarised before consensus; kappa will not be forced onto open-ended interpretive fields. Uncertainties will be resolved through consensus or third-reviewer adjudication. Substantive amendments will be recorded in the codebook change log and reported in the completed review.

The preliminary data charting form is provided in S2 Appendix.

### Data items

Data charting will focus on four domains: bibliographic characteristics, methodological characteristics, analytic process characteristics and reporting elements. Bibliographic characteristics will include author, year of publication, country or region, journal, disciplinary field, source type and, where applicable, feasibility-sampling status. Methodological characteristics will include the named concept analysis method, theoretical or methodological origin, rationale for method selection, type of evidence or data source, time frame of source material, search or source-identification procedures, assigned methodological lineage and procedural concordance with that lineage.

For this study, a methodological lineage is an identifiable concept-analysis tradition with a traceable methodological origin, recognisable core analytic logic or procedures, and expected outputs that permit recurrent sources to be mapped to the same tradition. A source that names and substantially follows a method will be assigned to that lineage. Adapted sources that retain the parent method’s core logic will remain within the parent lineage and be flagged as adapted. Sources that name a method but substantially depart from its core procedures will be retained descriptively as substantially modified but will not count toward lineage eligibility or lineage-level calculations. For combined approaches, a primary lineage will be assigned only when one method is clearly dominant and procedurally concordant or adapted; secondary methods will be flagged separately. Mixed sources without an identifiable primary lineage, and sources that cannot be reliably classified, will be retained in source-level mapping but excluded from lineage-level calculations. A derivative approach will be treated as a separate lineage only when it has a distinct traceable methodological origin and recurrent procedural logic; otherwise it will be coded as an adaptation of its parent lineage. An eligible methodological lineage is defined as a lineage represented by at least three included sources assigned as concordant or adapted before any feasibility sampling. Lineages represented by fewer than three such sources will be described as rare or emerging and will not enter lineage-level denominators.

Analytic process characteristics will include how analytic steps are presented; whether the source reports concept definitions, related or surrogate terms, attributes, antecedents, consequences, empirical referents, cases, context, temporality, fieldwork phases or conceptual diagrams. Reporting elements will include whether the title identifies concept analysis, whether the abstract reports the method, whether methods and analysis are transparently described, and whether limitations, ethics, reflexivity, implications and visual representations are reported.

Candidate reporting elements will be coded separately for occurrence, reporting adequacy or transparency, and conceptual applicability. Occurrence at the source level will be coded as Present, Absent, Not applicable or Unclear. Present indicates that the element is explicitly reported; Absent indicates that the element is applicable and evaluable but not reported; Not applicable indicates that the element is not logically relevant to the source type or methodological procedure; and Unclear indicates that the available report does not permit a reliable classification. Within each eligible methodological lineage, occurrence will be summarised as Present/(Present + Absent) among charted evaluable sources, with the numerator, denominator and percentage reported; Not applicable and Unclear will be reported separately and excluded from this denominator. Separate Sufficient/Partial/Insufficient ratings will be used only for designated methodological or reporting-transparency variables and will not substitute for occurrence coding.

Two empirical measures will be reported side by side. First, within-lineage occurrence will be expressed as the proportion of charted evaluable sources reporting the element in each eligible methodological lineage, as defined above. Second, cross-lineage breadth will be calculated as the number of evaluable eligible lineages with at least one charted source coded Present divided by the total number of evaluable eligible lineages; a lineage is evaluable when at least one charted source can be classified as Present or Absent. The two measures will be interpreted jointly: within-lineage occurrence describes how consistently an element appears among evaluable sources in a lineage, whereas cross-lineage breadth describes how many eligible lineages show any empirical presence. This dual-metric approach preserves the full within-lineage distribution rather than imposing an additional arbitrary within-lineage cut-off. For continuity with the prespecified protocol, an element may be described as widely observed when cross-lineage breadth is at least 70%; this descriptive label will always be presented with the corresponding within-lineage occurrence proportions and will not be interpreted as high prevalence within every lineage or as a universal, core or mandatory reporting item. Conceptual applicability will be judged independently of these empirical measures as cross-lineage, lineage-specific or unclear/contested. Cross-lineage applicability will require conceptual relevance to concept-analysis reporting irrespective of lineage-specific procedures, explicit methodological or empirical support in at least two eligible methodological lineages, and no identified lineage-specific incompatibility. Two reviewers will independently classify applicability; disagreements will be resolved by consensus and, if required, third-reviewer adjudication. The evidence basis and final decision will be retained in an audit log.

### Critical appraisal

Formal critical appraisal or risk-of-bias assessment of individual sources is not planned because the review will include heterogeneous methodological, theoretical and applied sources and aims to map methods and reporting elements rather than estimate effects or evidence certainty [15–17]. Instead, descriptive indicators of methodological and reporting transparency – including justification of method selection, source-identification procedures, analytic-step reporting, reflexivity and limitations – will be charted and considered when interpreting patterns. These indicators will not be combined into a quality score and will not be used to exclude, weight or rank sources.

### Data synthesis

The findings will be synthesised using descriptive summary, evidence mapping and deductive-inductive framework analysis [22]. The initial framework will contain six domains: source and disciplinary context; methodological lineage and philosophical orientation; evidence-source and identification procedures; analytic processes and outputs; reporting transparency; and candidate reporting-guidance status. The 66 candidate reporting items identified by Nashwan et al [12] will form the principal traceable deductive seed for the reporting-element codebook. Original item wording and source attribution will be retained; semantically overlapping items may be harmonised under normalised working labels while preserving links to the original items. Inductively identified elements may be added when the included evidence requires it. Inclusion in the initial deductive codebook will not imply that an item is universal, core, mandatory or cross-lineage applicable.

Analysis will proceed through familiarisation with included sources, dual pilot coding, revision and freezing of the initial codebook, matrix-based charting, within-lineage comparison, cross-lineage comparison, examination of deviant or contradictory cases, independent two-reviewer classification of candidate elements, and consensus or third-reviewer adjudication. Descriptive analyses will summarise publication year, geography, discipline, source type and distribution of methodological lineages. A methodological-lineage matrix will compare theoretical assumptions, data sources, analytic steps and outputs. A reporting-element matrix will present within-lineage occurrence proportions and cross-lineage breadth side by side, alongside the separate conceptual-applicability judgement defined above.

Primary visualisations will comprise a heatmap or matrix of methodological lineages by reporting elements, a lineage diagram showing relationships among identified methods, and descriptive bar charts for source and lineage distributions. An alluvial or Sankey diagram will be used only if the number and structure of transitions support an interpretable display. To manage interpretive influence, the review team will prepare a presupposition memo before formal coding, maintain reflexive analytic memos and a decision log, and discuss divergent interpretations in scheduled coding meetings. The completed review will distinguish findings extracted from included sources from the review team’s interpretive classifications.

### Data management and sharing

Search results, screening decisions, charting files, codebooks, analytic matrices and synthesis notes will be maintained in a version-controlled project structure. Read-only raw exports will be retained; editable files will use date-and-version identifiers; and a change log will record the editor, date, reason and affected variables. Each record will have a unique review ID linking screening, extraction and decision logs. Access will be restricted to the review team, with routine backup and a separate audit copy. No datasets have been generated for this protocol. On completion of the review, the final charting dataset, codebook, decision rules and analytic outputs will be made publicly available through the associated Open Science Framework project and/or the completed review’s Supporting Information.

### Ethical considerations

Ethical approval is not required because the review will analyse published and publicly accessible sources only and will not involve human participants, identifiable personal data or patient recruitment. Patients and members of the public were not involved in the design of this protocol because the review concerns published methodological and research sources rather than primary patient or service-user data.

### Study status and timeline

At the time of this revision in October 2026, the review remains at the protocol stage. Apart from pilot searches conducted on 5 May 2026 to test feasibility, sensitivity and platform syntax, no final database search, formal screening, full-text assessment, data charting or synthesis has begun. The final searches are planned for November 2026. Within the November 2026 to February 2027 review window, de-duplication and the prespecified title/abstract and full-text screening stages will precede preliminary lineage assignment and the 8–10-source charting pilot. The pilot will then inform the projected charting workload and the prespecified feasibility-contingency decision before formal charting begins; full or sampled charting, as applicable, will proceed through February 2027. This sequence preserves complete screening of the retrieved records while limiting any contingency sampling to the charting stage. Synthesis and preparation of the completed review manuscript are planned by May 2027. Any material change to this timeline will be recorded in the OSF amendment log and reported in the completed review.

### Protocol amendments

Any important amendment will be entered in a versioned OSF amendment log containing the date, protocol version, description, rationale, affected review stage and approving team members. The corresponding manuscript, search-strategy, codebook and charting-form versions will be cross-referenced. Amendments made after formal screening begins will additionally state whether previously screened or charted records were reassessed. All substantive amendments will be reported in the completed review.

## Discussion

This protocol sets out the methods for a scoping review of concept analysis lineages and their reporting elements across nursing and health-related disciplines. The review addresses a methodological problem that cannot be resolved by counting publications alone: occurrence frequency does not establish normative applicability, and different traditions may require different reporting expectations. The planned mapping will therefore report within-lineage occurrence and cross-lineage breadth as paired empirical measures while assessing cross-lineage or lineage-specific conceptual applicability separately. The review is an evidence-mapping and candidate-item generation stage, not a formal reporting-guideline development study.

The planned review has several strengths. It combines English- and Chinese-language databases, explicit calibration thresholds, duplicate screening, verified charting, a versioned codebook and deductive-inductive framework analysis. It will also make interpretive decisions auditable through independent classification, reflexive memos and a decision log. Treating reporting elements as linked to methodological traditions avoids assuming that features such as model cases, temporality, fieldwork phases or empirical referents are universally applicable.

Several limitations should be acknowledged. English- and Chinese-language restrictions may omit relevant sources in other languages. Grey literature will not be searched systematically, although targeted citation and author searches will seek foundational methodological books and chapters. Doctoral dissertations will be excluded to maintain the prespecified peer-reviewed publication boundary; this may underrepresent detailed applied concept analyses, particularly hybrid-model studies that include a fieldwork phase. An independent information-specialist PRESS review is not available; structured internal review against PRESS domains and seed-record testing will reduce, but cannot eliminate, search-design error. Formal critical appraisal will not be undertaken, so conclusions will address methodological and reporting patterns rather than source quality. Finally, neither high within-lineage occurrence nor broad cross-lineage presence can be treated as normatively essential without subsequent stakeholder appraisal and consensus.

Findings will be disseminated through peer-reviewed publication and academic presentations. The outputs may inform a future, separately governed reporting-guideline programme. If pursued, that programme would require stakeholder involvement, formal consensus methods such as a Delphi study, pilot testing, an explanation-and-elaboration document, and registration or engagement with the EQUATOR Network under a separate protocol.

## Supporting information

S1 Checklist PRISMA-P checklist. Completed PRISMA-P checklist for the protocol.
(DOCX)

S1 Appendix Search strategies for all databases. Pilot search strategies, provisional pre-deduplication counts, aggregate pilot yield, feasibility notes and the planned internal search-quality-control process for PubMed/MEDLINE, Embase, CINAHL, Scopus, Web of Science, PsycINFO, CNKI, Wanfang Data, VIP Information and China Biology Medicine.
(DOCX)

S2 Appendix Preliminary data charting form. Revised charting form, methodological-lineage assignment rules, candidate-item traceability, within-lineage occurrence and cross-lineage breadth coding, conceptual-applicability coding, calibration procedures, feasibility-sampling audit variables and version-control fields to be piloted before full charting.
(DOCX)

S1 Fig Planned PRISMA flow diagram. Planned study selection process for the scoping review.
(DOCX)
