# Supplementary material for: Methodological lineages of concept analysis and mapping of reporting elements in nursing and health-related disciplines: A scoping review protocol
Source: PLoS One. 2026 Oct 30;21(10):e0361727. doi: 10.1371/journal.pone.0361727 (PMC13722180; doi:10.1371/journal.pone.0361727)
Supplement: Supplementary file 2 [file pone.0361727.s002.docx]

**S1 Appendix. Supplemental material. Search strategies**

*Methodological lineages of concept analysis and mapping of reporting elements in nursing and health–related disciplines: a scoping review protocol*

## Search strategy notes

The tables below present pilot strategies for PubMed/MEDLINE, Embase, CINAHL, Scopus, Web of Science, PsycINFO, CNKI, Wanfang Data, VIP Information and China Biology Medicine. The Results column contains provisional counts from 5 May 2026. Across the final combination step in the ten databases, these provisional counts sum to 22,621 records before de–duplication. This aggregate is a cross–database retrieval count and is not a count of unique records, eligible sources or included sources. These are not final review yields. The final strategies will be quality–checked, versioned and re–run or updated before formal screening.

The strategy combines database–specific controlled vocabulary, where available, with fielded free–text terms for concept analysis phrases, named methodological lineages and nursing or health–related disciplinary context. Named–method terms remain primarily free text because most concept analysis lineages are not consistently indexed as controlled vocabulary. Title–only and major–topic restrictions will not be applied routinely because they may reduce sensitivity; proximity operators will be used only where supported and empirically helpful.

All records identified by the final searches will be de–duplicated and will undergo the prespecified title/abstract and full–text screening stages under the eligibility criteria. All eligible methodological, foundational, comparative and critical sources will be retained. The feasibility contingency specified in the manuscript and S2 Appendix will be considered only after full–text eligibility assessment and preliminary lineage assignment; it therefore does not reduce screening workload and is intended solely to reduce formal charting and verification burden. After the 8–10–source charting pilot, the observed charting–and–verification time will be extrapolated to the full set of eligible applied concept analyses. Sampling will be activated if the projected charting–and–verification workload exceeds 160 combined reviewer–hours, corresponding to the prespecified maximum reviewer capacity allocated to formal charting and verification within the planned review timeline. If activated, 50% of eligible applied concept analyses within each eligible methodological lineage will be selected by reproducible lineage–stratified random sampling, rounded up to the next whole source. At least three eligible applied concept analyses will be retained within each lineage where three or more are available; if fewer than three are available, all will be retained. Sources in rare or emerging lineages will be retained in full. The sampling frame, lineage allocation, sampling fraction, random seed and rationale will be frozen and documented in the OSF amendment log before sampled charting begins. Within the November 2026 to February 2027 review window, screening and preliminary lineage assignment will precede the charting pilot, workload projection, contingency decision and subsequent full or sampled charting.

Before final execution, two review–team members who did not draft the initial strategies will independently check the PubMed strategy and all database translations against the PRESS 2015 domains as an internal quality–control checklist. This process is not formal PRESS peer review by an independent information specialist. The reviewers will check subject headings and text words, spelling and syntax, Boolean and proximity operators, line combinations, limits, database translations and known seed–source recall. All changes will be recorded in a dated search–version log.

## Table S1. Search strategy for PubMed/MEDLINE

| **#Step** | **Search terms and Boolean logic** | **Results** |
| --- | --- | --- |
| 1 | "concept analysis"[Title/Abstract] OR "concept analyses"[Title/Abstract] OR "conceptual analysis"[Title/Abstract] OR "concept clarification"[Title/Abstract] OR "concept development"[Title/Abstract] OR "concept derivation"[Title/Abstract] OR "concept synthesis"[Title/Abstract] OR "evolutionary concept analysis"[Title/Abstract] OR "hybrid concept analysis"[Title/Abstract] | 4,796 |
| 2 | ("Walker and Avant"[Title/Abstract] OR "Walker & Avant"[Title/Abstract] OR Rodgers[Title/Abstract] OR "Rodgers evolutionary"[Title/Abstract] OR "hybrid model"[Title/Abstract] OR "principle–based concept analysis"[Title/Abstract] OR "pragmatic utility"[Title/Abstract] OR "simultaneous concept analysis"[Title/Abstract] OR "semantic concept analysis"[Title/Abstract]) AND (concept*[Title/Abstract] OR "concept analysis"[Title/Abstract]) | 2,016 |
| 3 | #1 OR #2 | 5,087 |
| 4 | "Nursing"[MeSH Terms] OR "Nursing Research"[MeSH Terms] OR "Nursing Care"[MeSH Terms] OR "Education, Nursing"[MeSH Terms] OR "Nursing Services"[MeSH Terms] OR "Nursing Staff"[MeSH Terms] OR "Allied Health Personnel"[MeSH Terms] OR "Rehabilitation"[MeSH Terms] OR "Public Health"[MeSH Terms] OR nurs*[Title/Abstract] OR midwif*[Title/Abstract] OR healthcare[Title/Abstract] OR "health care"[Title/Abstract] OR "health services"[Title/Abstract] OR "health services research"[Title/Abstract] OR "health science"[Title/Abstract] OR "health sciences"[Title/Abstract] OR "allied health"[Title/Abstract] OR rehabilitat*[Title/Abstract] OR "public health"[Title/Abstract] | 11,903,242 |
| 5 | #3 AND #4 | 3,574 |
| Filters | Pilot run limited to English and Chinese, with coverage from database inception to 5 May 2026. The final strategy will be quality–checked, versioned and re–run or updated before formal screening; final dates, limits and counts will replace the pilot information in the completed review. | – |

## Table S2. Search strategy for Embase

| **#Step** | **Search terms and Boolean logic** | **Results** |
| --- | --- | --- |
| 1 | 'concept analysis':ti,ab,kw OR 'concept analyses':ti,ab,kw OR 'conceptual analysis':ti,ab,kw OR 'concept clarification':ti,ab,kw OR 'concept development':ti,ab,kw OR 'concept derivation':ti,ab,kw OR 'concept synthesis':ti,ab,kw OR 'evolutionary concept analysis':ti,ab,kw OR 'hybrid concept analysis':ti,ab,kw | 5,068 |
| 2 | ('walker and avant':ti,ab,kw OR 'walker & avant':ti,ab,kw OR rodgers:ti,ab,kw OR 'rodgers evolutionary':ti,ab,kw OR 'hybrid model':ti,ab,kw OR 'principle–based concept analysis':ti,ab,kw OR 'pragmatic utility':ti,ab,kw OR 'simultaneous concept analysis':ti,ab,kw OR 'semantic concept analysis':ti,ab,kw) AND concept*:ti,ab,kw | 1,967 |
| 3 | #1 OR #2 | 5,386 |
| 4 | 'nursing'/exp OR 'nurse'/exp OR 'midwife'/exp OR 'rehabilitation'/exp OR 'public health'/exp OR nurs*:ti,ab,kw OR midwif*:ti,ab,kw OR healthcare:ti,ab,kw OR 'health care':ti,ab,kw OR 'health service':ti,ab,kw OR 'health services':ti,ab,kw OR 'health services research':ti,ab,kw OR 'health science':ti,ab,kw OR 'health sciences':ti,ab,kw OR 'allied health':ti,ab,kw OR rehabilitat*:ti,ab,kw OR 'public health':ti,ab,kw | 3,907,932 |
| 5 | #3 AND #4 | 3,066 |
| Filters | Pilot run limited to English and Chinese, with coverage from database inception to 5 May 2026. The final strategy will be quality–checked, versioned and re–run or updated before formal screening; final dates, limits and counts will replace the pilot information in the completed review. Platform syntax shown for Embase.com/Elsevier; adapt if searched through Ovid. | – |

## Table S3. Search strategy for CINAHL via EBSCOhost

| **#Step** | **Search terms and Boolean logic** | **Results** |
| --- | --- | --- |
| 1 | TI ("concept analysis" OR "concept analyses" OR "conceptual analysis" OR "concept clarification" OR "concept development" OR "concept derivation" OR "concept synthesis" OR "evolutionary concept analysis" OR "hybrid concept analysis") OR AB ("concept analysis" OR "concept analyses" OR "conceptual analysis" OR "concept clarification" OR "concept development" OR "concept derivation" OR "concept synthesis" OR "evolutionary concept analysis" OR "hybrid concept analysis") | 3,978 |
| 2 | (TI ("Walker and Avant" OR "Walker & Avant" OR Rodgers OR "Rodgers evolutionary" OR "hybrid model" OR "principle–based concept analysis" OR "pragmatic utility" OR "simultaneous concept analysis" OR "semantic concept analysis") OR AB ("Walker and Avant" OR "Walker & Avant" OR Rodgers OR "Rodgers evolutionary" OR "hybrid model" OR "principle–based concept analysis" OR "pragmatic utility" OR "simultaneous concept analysis" OR "semantic concept analysis")) AND (TI concept* OR AB concept*) | 2,163 |
| 3 | #1 OR #2 | 4,921 |
| 4 | MH "Nursing+" OR MH "Nurses+" OR MH "Midwifery+" OR MH "Rehabilitation+" OR MH "Public Health+" OR TI nurs* OR AB nurs* OR TI midwif* OR AB midwif* OR TI healthcare OR AB healthcare OR TI "health care" OR AB "health care" OR TI "health services" OR AB "health services" OR TI "health services research" OR AB "health services research" OR TI "health science" OR AB "health science" OR TI "health sciences" OR AB "health sciences" OR TI "allied health" OR AB "allied health" OR TI rehabilitat* OR AB rehabilitat* OR TI "public health" OR AB "public health" | 2,893,244 |
| 5 | #3 AND #4 | 3,436 |
| Filters | Pilot run limited to English and Chinese, with coverage from database inception to 5 May 2026. The final strategy will be quality–checked, versioned and re–run or updated before formal screening; final dates, limits and counts will replace the pilot information in the completed review. Platform: EBSCOhost. | – |

## Table S4. Search strategy for Scopus

| **#Step** | **Search terms and Boolean logic** | **Results** |
| --- | --- | --- |
| 1 | TITLE–ABS–KEY ( "concept analysis" OR "concept analyses" OR "conceptual analysis" OR "concept clarification" OR "concept development" OR "concept derivation" OR "concept synthesis" OR "evolutionary concept analysis" OR "hybrid concept analysis" ) | 24,916 |
| 2 | TITLE–ABS–KEY ( "Walker and Avant" OR "Walker & Avant" OR Rodgers OR "Rodgers evolutionary" OR "hybrid model" OR "principle–based concept analysis" OR "pragmatic utility" OR "simultaneous concept analysis" OR "semantic concept analysis" ) AND TITLE–ABS–KEY ( concept* ) | 4,940 |
| 3 | #1 OR #2 | 27,711 |
| 4 | TITLE–ABS–KEY ( nurs* OR midwif* OR healthcare OR "health care" OR "health services" OR "health services research" OR "health science" OR "health sciences" OR "allied health" OR rehabilitat* OR "public health" ) | 5,136,507 |
| 5 | #3 AND #4 | 4,663 |
| Filters | Pilot run limited to English and Chinese, with coverage from database inception to 5 May 2026. The final strategy will be quality–checked, versioned and re–run or updated before formal screening; final dates, limits and counts will replace the pilot information in the completed review. Apply language and date limits using Scopus filters after running the search. | – |

## Table S5. Search strategy for Web of Science Core Collection

| **#Step** | **Search terms and Boolean logic** | **Results** |
| --- | --- | --- |
| 1 | TS=("concept analysis" OR "concept analyses" OR "conceptual analysis" OR "concept clarification" OR "concept development" OR "concept derivation" OR "concept synthesis" OR "evolutionary concept analysis" OR "hybrid concept analysis") | 12,836 |
| 2 | TS=(("Walker and Avant" OR "Walker & Avant" OR Rodgers OR "Rodgers evolutionary" OR "hybrid model" OR "principle–based concept analysis" OR "pragmatic utility" OR "simultaneous concept analysis" OR "semantic concept analysis") AND concept*) | 2,735 |
| 3 | #1 OR #2 | 14,147 |
| 4 | TS=(nurs* OR midwif* OR healthcare OR "health care" OR "health services" OR "health services research" OR "health science" OR "health sciences" OR "allied health" OR rehabilitat* OR "public health") | 2,266,365 |
| 5 | #3 AND #4 | 2,564 |
| Filters | Pilot run limited to English and Chinese, with coverage from database inception to 5 May 2026. The final strategy will be quality–checked, versioned and re–run or updated before formal screening; final dates, limits and counts will replace the pilot information in the completed review. Database: Web of Science Core Collection. | – |

## Table S6. Search strategy for PsycINFO via EBSCOhost

| **#Step** | **Search terms and Boolean logic** | **Results** |
| --- | --- | --- |
| 1 | TI ("concept analysis" OR "concept analyses" OR "conceptual analysis" OR "concept clarification" OR "concept development" OR "concept derivation" OR "concept synthesis" OR "evolutionary concept analysis" OR "hybrid concept analysis") OR AB ("concept analysis" OR "concept analyses" OR "conceptual analysis" OR "concept clarification" OR "concept development" OR "concept derivation" OR "concept synthesis" OR "evolutionary concept analysis" OR "hybrid concept analysis") | 4,376 |
| 2 | (TI ("Walker and Avant" OR "Walker & Avant" OR Rodgers OR "Rodgers evolutionary" OR "hybrid model" OR "principle–based concept analysis" OR "pragmatic utility" OR "simultaneous concept analysis" OR "semantic concept analysis") OR AB ("Walker and Avant" OR "Walker & Avant" OR Rodgers OR "Rodgers evolutionary" OR "hybrid model" OR "principle–based concept analysis" OR "pragmatic utility" OR "simultaneous concept analysis" OR "semantic concept analysis")) AND (TI concept* OR AB concept*) | 699 |
| 3 | #1 OR #2 | 4,595 |
| 4 | DE "Nursing" OR TI nurs* OR AB nurs* OR TI midwif* OR AB midwif* OR TI healthcare OR AB healthcare OR TI "health care" OR AB "health care" OR TI "health services" OR AB "health services" OR TI "health services research" OR AB "health services research" OR TI "health science" OR AB "health science" OR TI "health sciences" OR AB "health sciences" OR TI "allied health" OR AB "allied health" OR TI rehabilitat* OR AB rehabilitat* OR TI "public health" OR AB "public health" | 580,781 |
| 5 | #3 AND #4 | 1,145 |
| Filters | Pilot run limited to English and Chinese, with coverage from database inception to 5 May 2026. The final strategy will be quality–checked, versioned and re–run or updated before formal screening; final dates, limits and counts will replace the pilot information in the completed review. Platform: EBSCOhost; adapt controlled vocabulary if searched via APA PsycNet or Ovid. | – |

## Table S7. Search strategy for CNKI (China National Knowledge Infrastructure)

| **#Step** | **Search terms and Boolean logic** | **Results** |
| --- | --- | --- |
| 1 | SU=("概念分析" OR "概念分析法" OR "概念分析方法" OR "概念界定" OR "概念澄清" OR "概念开发" OR "概念发展" OR "概念衍生" OR "概念合成" OR "演化概念分析" OR "混合模型" OR "混合式概念分析" OR "Walker" OR "Avant" OR "Rodgers" OR "罗杰斯") English translation: concept analysis OR concept analysis method OR concept definition/clarification/development/derivation/synthesis OR evolutionary concept analysis OR hybrid model OR named methods/authors. | 54,543 |
| 2 | SU=("护理" OR "护理学" OR "护士" OR "临床护理" OR "护理教育" OR "护理管理" OR "助产" OR "康复" OR "公共卫生" OR "健康照护" OR "医疗卫生" OR "卫生服务") English translation: nursing OR nursing science OR nurse OR clinical nursing OR nursing education OR nursing management OR midwifery OR rehabilitation OR public health OR health care OR health services. | 1,951,212 |
| 3 | #1 AND #2 | 879 |
| Filters | Pilot run limited to Chinese and English, with coverage from database inception to 5 May 2026. The final strategy will be quality–checked, versioned and re–run or updated before formal screening; final dates, limits and counts will replace the pilot information in the completed review. Apply source–type limits according to final eligibility criteria. | – |

## Table S8. Search strategy for Wanfang Data

| **#Step** | **Search terms and Boolean logic** | **Results** |
| --- | --- | --- |
| 1 | 主题:("概念分析" OR "概念分析法" OR "概念分析方法" OR "概念界定" OR "概念澄清" OR "概念开发" OR "概念发展" OR "概念衍生" OR "概念合成" OR "演化概念分析" OR "混合模型" OR "混合式概念分析" OR "Walker" OR "Avant" OR "Rodgers" OR "罗杰斯") English translation: concept analysis OR concept analysis method OR concept definition/clarification/development/derivation/synthesis OR evolutionary concept analysis OR hybrid model OR named methods/authors. | 149,727 |
| 2 | 主题:("护理" OR "护理学" OR "护士" OR "临床护理" OR "护理教育" OR "护理管理" OR "助产" OR "康复" OR "公共卫生" OR "健康照护" OR "医疗卫生" OR "卫生服务") English translation: nursing OR nursing science OR nurse OR clinical nursing OR nursing education OR nursing management OR midwifery OR rehabilitation OR public health OR health care OR health services. | 3,969,091 |
| 3 | #1 AND #2 | 2,476 |
| Filters | Pilot run limited to Chinese and English, with coverage from database inception to 5 May 2026. The final strategy will be quality–checked, versioned and re–run or updated before formal screening; final dates, limits and counts will replace the pilot information in the completed review. Apply source–type limits according to final eligibility criteria. | – |

## Table S9. Search strategy for VIP Information (VIP Database)

| #Step | Search terms and Boolean logic | Results |
| --- | --- | --- |
| 1 | M=("概念分析" OR "概念分析法" OR "概念分析方法" OR "概念界定" OR "概念澄清" OR "概念开发" OR "概念发展" OR "概念衍生" OR "概念合成" OR "演化概念分析" OR "混合模型" OR "混合式概念分析" OR "Walker" OR "Avant" OR "Rodgers" OR "罗杰斯") M indicates title/keyword/abstract/common search fields in VIP, depending on platform display. | 21,749 |
| 2 | M=("护理" OR "护理学" OR "护士" OR "临床护理" OR "护理教育" OR "护理管理" OR "助产" OR "康复" OR "公共卫生" OR "健康照护" OR "医疗卫生" OR "卫生服务") | 1,793,357 |
| 3 | #1 AND #2 | 258 |
| Filters | Pilot run limited to Chinese and English, with coverage from database inception to 5 May 2026. The final strategy will be quality–checked, versioned and re–run or updated before formal screening; final dates, limits and counts will replace the pilot information in the completed review. Apply source–type limits according to final eligibility criteria. M indicates title/keyword/abstract/common search fields in VIP, depending on platform display. | – |

## Table S10. Search strategy for China Biology Medicine (CBM/SinoMed)

| **#Step** | **Search terms and Boolean logic** | **Results** |
| --- | --- | --- |
| 1 | "概念分析"[常用字段] OR "概念分析法"[常用字段] OR "概念分析方法"[常用字段] OR "概念界定"[常用字段] OR "概念澄清"[常用字段] OR "概念开发"[常用字段] OR "概念发展"[常用字段] OR "概念衍生"[常用字段] OR "概念合成"[常用字段] OR "演化概念分析"[常用字段] OR "混合模型"[常用字段] OR "混合式概念分析"[常用字段] OR "Walker"[常用字段] OR "Avant"[常用字段] OR "Rodgers"[常用字段] OR "罗杰斯"[常用字段] | 20,431 |
| 2 | "护理"[常用字段] OR "护理学"[常用字段] OR "护士"[常用字段] OR "临床护理"[常用字段] OR "护理教育"[常用字段] OR "护理管理"[常用字段] OR "助产"[常用字段] OR "康复"[常用字段] OR "公共卫生"[常用字段] OR "健康照护"[常用字段] OR "医疗卫生"[常用字段] OR "卫生服务"[常用字段] | 3,451,502 |
| 3 | #1 AND #2 | 560 |
| Filters | Pilot run limited to Chinese and English, with coverage from database inception to 5 May 2026. The final strategy will be quality–checked, versioned and re–run or updated before formal screening; final dates, limits and counts will replace the pilot information in the completed review. Apply source–type limits according to final eligibility criteria. | – |

## References for search design and reporting

Peters MDJ, Marnie C, Tricco AC, et al. Updated methodological guidance for the conduct of scoping reviews. JBI Evid Synth. 2020;18(10):2119–2126.

Peters MDJ, Godfrey C, McInerney P, et al. Best practice guidance and reporting items for the development of scoping review protocols. JBI Evid Synth. 2022;20(4):953–968.

Tricco AC, Lillie E, Zarin W, et al. PRISMA extension for scoping reviews (PRISMA–ScR): Checklist and explanation. Ann Intern Med. 2018;169(7):467–473.

National Library of Medicine. Medical Subject Headings (MeSH).

EBSCO. CINAHL database and CINAHL subject headings guidance.

Elsevier. Embase search guidance and Emtree syntax.

McGowan J, Sampson M, Salzwedel DM, et al. PRESS Peer Review of Electronic Search Strategies: 2015 guideline statement. J Clin Epidemiol. 2016;75:40–46.

Rethlefsen ML, Kirtley S, Waffenschmidt S, et al. PRISMA–S: an extension to the PRISMA Statement for reporting literature searches in systematic reviews. Syst Rev. 2021;10:39.
