# Supplementary material for: Methodological lineages of concept analysis and mapping of reporting elements in nursing and health-related disciplines: A scoping review protocol
Source: PLoS One. 2026 Oct 30;21(10):e0361727. doi: 10.1371/journal.pone.0361727 (PMC13722180; doi:10.1371/journal.pone.0361727)
Supplement: Supplementary file 3 [file pone.0361727.s003.docx]

**S2 Appendix. Preliminary data charting form**

# **Purpose:** This preliminary data charting form will be used in the planned scoping review on methodological lineages of concept analysis and reporting elements in nursing and health–related disciplines. The form will be piloted and refined before full data charting.

# Instructions

- Chart data from each included source using the variables listed below.
- Use verbatim extraction for key methodological descriptions where appropriate.
- For candidate reporting–element occurrence, use Present, Absent, Not applicable or Unclear according to the operational rules below. For other variables, use NA when a variable is not applicable and Unclear when information cannot be determined from the source.
- Record uncertainties in the notes column for team discussion.
- Document substantive revisions to the charting form during the review process.

| **Item** | **Variable** | **Description** | **Response options or coding** |
| --- | --- | --- | --- |
| **Section A. Bibliographic characteristics** | | | |
| A1 | Record ID | Unique review identification number | Free text |
| A2 | Full citation | Author(s), year, title, journal or source | Free text |
| A3 | Year of publication | Year in which the source was published | Numerical |
| A4 | Country or region | Country/region of first author or study context, where available | Free text |
| A5 | Journal or source | Journal, database, book or other source title | Free text |
| A6 | Disciplinary field | Nursing, midwifery, rehabilitation, public health, allied health, health services research or other | Predefined categories plus free text |
| A7 | Source type | Methodological paper, applied concept analysis study, comparative/critical discussion paper or other scholarly source | Categorical |
| A8 | Language | Language of publication | English / Chinese / other |
| A9 | Applied–analysis sampling status | Audit status after applying the prespecified charting–workload activation rule and, if activated, the prespecified sampling fraction | Not applicable (methodological/foundational/comparative–critical source) / Retained in full / Eligible for stratified sampling / Randomly sampled / Not selected |
| A10 | Sampling stratum and randomisation record | Lineage stratum, sampling–frame identifier, prespecified sampling fraction and reproducible randomisation information if contingency sampling is activated | Lineage label / sampling–frame ID / sampling fraction / random seed or random rank / NA if contingency not activated |
| **Section B. Methodological characteristics** | | | |
| B1 | Named concept analysis method | Named method used or discussed in the source | Walker and Avant / Rodgers / hybrid model / principle–based / pragmatic / other / unclear |
| B1a | Assigned methodological lineage | Normalised lineage assigned after considering the named method, methodological origin and actual procedures | Normalised lineage label / Unclear–unclassifiable |
| B1b | Procedural concordance | Degree to which the source follows the core logic or procedures of the assigned lineage | Concordant / Adapted / Substantially modified / Unclear |
| B1c | Mixed, combined or derivative status | Whether more than one approach is combined or the approach is explicitly derivative/adapted | None / Mixed with identifiable primary lineage / Mixed without primary lineage / Derivative–adapted / Other |
| B1d | Primary and secondary method note | Primary lineage and any secondary method(s) used in a combined source | Free text / NA |
| B1e | Lineage eligibility status | Lineage–level status determined from the full eligible source set before any feasibility sampling; only concordant or adapted sources count toward the threshold | Eligible lineage (at least 3 assignable sources) / Rare–emerging lineage (<3) / Not eligible for lineage–level calculation |
| B2 | Original methodological source cited | Whether the original or key methodological source is cited | Yes / No / Unclear; citation note |
| B3 | Rationale for method selection | Whether authors justify selection of the method | Yes / No / Partial / Unclear |
| B4 | Philosophical or theoretical orientation | Whether the source explains the underlying orientation of the method | Yes / No / Unclear; extract if yes |
| B5 | Evidence or data source type | Sources used for the concept analysis | Literature only / literature plus fieldwork / mixed / other / unclear |
| B6 | Search or source–identification process | Whether searching or source identification is described | Yes / No / Partial |
| B7 | Time frame of source material | Whether a time period for source material is reported | Yes / No / Unclear |
| **Section C. Analytic process characteristics** | | | |
| C1 | Analytic steps | Whether analytic steps are described | Yes / No / Partial |
| C2 | Final concept definition | Whether a final or synthesised definition is reported | Yes / No |
| C3 | Related or surrogate concepts | Whether related, surrogate or neighbouring concepts are reported | Yes / No |
| C4 | Attributes or defining characteristics | Whether attributes or defining characteristics are reported | Yes / No |
| C5 | Antecedents | Whether antecedents are reported | Yes / No |
| C6 | Consequences | Whether consequences are reported | Yes / No |
| C7 | Empirical referents or observable indicators | Whether empirical referents or observable indicators are reported | Yes / No / Not applicable |
| C8 | Cases | Whether cases are reported and their type | No cases / model / borderline / contrary / related / invented / illegitimate / other / type unclear |
| C9 | Diagram or conceptual model | Whether a visual representation is included | Yes / No |
| C10 | Context or temporality | Whether context, temporality or evolution of the concept is discussed | Yes / No / Unclear |
| C11 | Fieldwork phase | Whether a fieldwork phase is included | Yes / No / Not applicable |
| **Section D. Descriptive reporting and transparency indicators (separate from candidate–element occurrence coding)** | | | |
| D1 | Title identifies concept analysis | Whether the title explicitly identifies the study as concept analysis | Yes / No |
| D2 | Abstract states method | Whether the abstract reports the methodological approach | Yes / No / Partial |
| D3 | Aim or objective clearly stated | Whether the purpose of the concept analysis is clear | Yes / No |
| D4 | Method transparency | Whether the method is described sufficiently for readers to understand what was done | Sufficient / Partial / Insufficient |
| D5 | Data source transparency | Whether data sources are clearly reported | Sufficient / Partial / Insufficient |
| D6 | Analytic transparency | Whether the analytic process is clearly reported | Sufficient / Partial / Insufficient |
| D7 | Reflexivity | Whether author reflexivity or interpretive positioning is discussed | Yes / No |
| D8 | Ethical considerations | Whether ethical considerations are discussed, if applicable | Yes / No / Not applicable |
| D9 | Limitations | Whether limitations are discussed | Yes / No |
| D10 | Implications or recommendations | Whether implications for theory, research, education, policy or practice are discussed | Yes / No |
| D11 | Visual representation | Whether a diagram, model or other visual representation is provided | Yes / No |
| **Section E. Candidate reporting–element occurrence, distribution and applicability** | | | |
| E1 | Candidate reporting element ID and normalised label | Unique working identifier and normalised label for the candidate reporting element | Candidate–item ID / normalised label |
| E2 | Origin and traceability | Trace the candidate item to the initial deductive pool or to an inductive source | Nashwan et al. item ID + original wording/source / Inductive item + source |
| E3 | Harmonisation status | How wording overlap or duplication has been handled without losing original–item traceability | Unchanged / Normalised wording / Merged with item ID(s) / Split / Inductively added |
| E4 | Source–level occurrence code | Whether the candidate reporting element is reported in the source | Present / Absent / Not applicable / Unclear |
| E5 | Occurrence evidence note | Verbatim extract, location or summary supporting the occurrence code and any uncertainty | Quotation / page or section / reviewer note |
| E6 | Applicability classification | Conceptual applicability judged independently of empirical frequency | Cross–lineage / Lineage–specific / Unclear or contested |
| E7 | Within–lineage occurrence proportion | Occurrence among charted evaluable sources within each eligible methodological lineage; reported as the first empirical measure | Present / (Present + Absent); report numerator, denominator and percentage; Not applicable and Unclear excluded and reported separately |
| E8 | Lineage evaluability and empirical presence | A lineage is evaluable when at least one charted source is Present or Absent; empirical presence for the breadth measure requires at least one Present source | Evaluable–present / Evaluable–no presence / Not evaluable |
| E9 | Cross–lineage breadth | Breadth of empirical presence across evaluable eligible methodological lineages; reported side by side with within–lineage occurrence proportions | Evaluable eligible lineages with at least one Present source / all evaluable eligible lineages; report numerator, denominator and percentage |
| E10 | Widely observed descriptor | Descriptive cross–lineage breadth label only; always interpreted with within–lineage occurrence proportions and does not define a universal, core or mandatory item | Yes if cross–lineage breadth is >=70% / No / Not calculable |
| E11 | Conceptual support | Methodological or empirical basis for applicability classification; cross–lineage requires conceptual relevance irrespective of lineage–specific procedures, support in at least two eligible lineages, and no identified lineage–specific incompatibility | Citation(s) / source–level evidence / analytic memo |
| E12 | Reviewer 1 judgement | Independent applicability judgement before consensus | Cross–lineage / Lineage–specific / Unclear or contested |
| E13 | Reviewer 2 judgement | Independent applicability judgement before consensus | Cross–lineage / Lineage–specific / Unclear or contested |
| E14 | Consensus and adjudication | Final applicability decision, rationale and any third–reviewer involvement | Decision text / rationale / adjudicator / date |
| **Section F. Audit and version control** | | | |
| F1 | Codebook version | Codebook version used for the record | Version number and date |
| F2 | Decision–log ID | Identifier linking uncertainties and consensus decisions | Unique ID |
| F3 | Reflexive memo | Review–team presupposition or interpretive influence relevant to coding | Memo or linked memo ID |
| F4 | Amendment status | Whether the record was reassessed following a protocol or codebook amendment | No / Yes; version and date |

# **Calibration plan:** Before title and abstract screening, two reviewers will independently assess a purposively enriched set of 25 records containing likely inclusions, likely exclusions and borderline or uncertain records. Formal screening will begin after both overall raw agreement and positive agreement for the include/uncertain decision reach at least 75%; otherwise, the eligibility criteria will be clarified and calibration repeated with a new enriched set. Positive agreement will be calculated before consensus for the binary include/uncertain versus exclude decision as twice the number of records classified include/uncertain by both reviewers divided by the sum of all include/uncertain classifications made by the two reviewers; Cohen's kappa will also be calculated and interpreted descriptively rather than used as the sole screening gate. Before full–text screening, two reviewers will independently assess 5–10 purposively selected boundary cases. Before full charting, two reviewers will independently pilot this form on 8–10 purposively selected sources representing different concept analysis lineages and source types. They will compare categorical and free–text data, refine definitions, document disagreements and freeze codebook version 1.0 before formal charting. Agreement on categorical charting variables will be summarised before consensus; free–text fields will be reconciled through source verification and discussion rather than forced kappa calculation.

# Feasibility contingency: All records will complete the prespecified title/abstract and full–text screening process, and all eligible methodological, foundational, comparative and critical sources will be retained. The feasibility contingency will be considered only after full–text eligibility assessment and preliminary lineage assignment; it therefore reduces only formal charting and verification burden, not screening workload. During the 8–10–source charting pilot, the charting–and–verification time will be recorded and extrapolated to the full set of eligible applied concept analyses. Sampling will be activated if the projected charting–and–verification workload exceeds 160 combined reviewer–hours, corresponding to the prespecified maximum reviewer capacity allocated to formal charting and verification within the planned review timeline. If activated, 50% of eligible applied concept analyses within each eligible methodological lineage will be selected by reproducible lineage–stratified random sampling, rounded up to the next whole source. At least three eligible applied concept analyses will be retained within each lineage where three or more are available; if fewer than three are available, all will be retained. Sources in rare or emerging lineages will be retained in full. The sampling frame, lineage allocation, sampling fraction, random seed and rationale will be frozen in the OSF amendment log before sampled charting begins. Lineage eligibility will be determined from the full eligible source set before sampling, and no reporting–element data will be used to determine sampling. Within the November 2026 to February 2027 review window, screening and preliminary lineage assignment will precede the charting pilot, workload projection, contingency decision and subsequent full or sampled charting.

# Operational coding rules: A methodological lineage is an identifiable concept–analysis tradition with a traceable methodological origin, recognisable core analytic logic or procedures and expected outputs. Named–method sources that are procedurally concordant are assigned to that lineage; adapted sources that retain the parent method's core logic remain in the parent lineage and are flagged as adapted. Substantially modified sources are retained descriptively but do not count toward lineage eligibility or lineage–level calculations. Mixed or combined sources are assigned a primary lineage only when one method is clearly dominant and concordant or adapted; otherwise they are coded mixed/combined with no primary lineage and retained only for source–level mapping. Unclear or unclassifiable sources are handled similarly. A derivative approach is treated as a separate lineage only when it has a distinct traceable origin and recurrent procedural logic; otherwise it is treated as an adaptation of the parent lineage. An eligible methodological lineage requires at least three included sources assigned as concordant or adapted before any feasibility sampling. Candidate reporting–element occurrence is coded Present, Absent, Not applicable or Unclear; adequacy/transparency is coded separately where specified. Empirical distribution will be reported using two measures side by side: within–lineage occurrence proportion [Present/(Present + Absent)] and cross–lineage breadth (evaluable eligible lineages with at least one Present source divided by all evaluable eligible lineages). The widely observed descriptor, when cross–lineage breadth is at least 70%, is descriptive only and will always be interpreted with the within–lineage proportions. Conceptual applicability is classified independently as Cross–lineage, Lineage–specific or Unclear/contested.

# Amendments and reflexivity: Each substantive change will be recorded with date, version, rationale, affected variables and approving reviewers. Before formal coding, reviewers will record methodological presuppositions; reflexive memos, unresolved questions and adjudication decisions will be linked to the relevant record and codebook version.
