# Supplementary material for: Methodological lineages of concept analysis and mapping of reporting elements in nursing and health-related disciplines: A scoping review protocol
Source: PLoS One. 2026 Oct 30;21(10):e0361727. doi: 10.1371/journal.pone.0361727 (PMC13722180; doi:10.1371/journal.pone.0361727)
Supplement: Supplementary file 4 [file pone.0361727.s004.docx]

**Identification of records via supplementary methods**

**Identification of studies via databases**

Records identified from:

Databases (n = )

PubMed/MEDLINE (n = )

Embase (n = )

CINAHL (n = )

Scopus (n = )

Web of Science (n = )

PsycINFO (n = )

CNKI (n = )

Wanfang Data (n = )

VIP Information (n = )

SinoMed/CBM (n = )

Records identified from:

Supplementary methods (n = )

Backward citation searching (n = )

Forward citation tracking (n = )

Reference list screening (n = )

Records removed *before screening*:

Duplicate records removed (n = )

**Identification**

Records excluded

(n = )

**Screening**

Title and abstracts screening

(n = )

Reports not retrieved

(n = )

Records sought retrieval

(n = )

Reports not retrieved

(n = )

Full-text articles sought for retrieval (n = )

Reports excluded:

Reason 1 (n = )

Reason 2 (n = )

Reason 3 (n = )

etc.

Full-text articles assessed for eligibility (n = )

Reports excluded:

Reason 1 (n = )

Reason 2 (n = )

Reason 3 (n = )

etc.

Records assessed for eligibility (n = )

Sources included in the scoping review

**Included**
